# Matrix stiffness modulates hepatic stellate cell activation into tumor-promoting myofibroblasts via E2F3-dependent signaling and regulates malignant progression

**DOI:** 10.1038/s41419-021-04418-9

**Published:** 2021-12-06

**Authors:** Zhikui Liu, Huanye Mo, Runkun Liu, Yongshen Niu, Tianxiang Chen, Qiuran Xu, Kangsheng Tu, Nan Yang

**Affiliations:** 1 Department of Hepatobiliary Surgery, the First Affiliated Hospital of Xi’an Jiaotong University, No. 277 Yanta West Road, Xi’an, 710061 China; 2 Key Laboratory of Tumor Molecular Diagnosis and Individualized Medicine of Zhejiang Province, Zhejiang Provincial People’s Hospital (People’s Hospital of Hangzhou Medical College), Hangzhou, Zhejiang 310014 China; 3 Department of Infectious Diseases, the First Affiliated Hospital of Xi’an Jiaotong University, No. 277 Yanta West Road, Xi’an, 710061 China

**Keywords:** Liver cancer, Mechanisms of disease

## Abstract

The hepatic stellate cells (HSCs) activation by myofibroblastic differentiation is critical for liver fibrosis. Crosstalk between stromal cells and tumor cells in the microenvironment alters the properties and facilitates the growth and metastasis of tumor cells. How mechanical stimuli originally stiffness of extracellular matrix (ECM) contribute to tumor development remains poorly understood. Here, we demonstrated that stiffness contributes to mechanosignal transduction in HSCs, which promotes hepatocellular carcinoma (HCC) cells growth and metastasis through secretion of FGF2. On stiffness matrix, HSCs activation was confirmed by immunofluorescence (IF) and Western blot (WB) for α-smooth muscle actin (SMA). Increasing matrix stiffness promoted HSCs activation by CD36-AKT-E2F3 mechanosignaling through shRNA-mediated E2F3 knockdown, AKT inhibitors, and CD36 shRNA. Moreover, ChIP-qPCR. Confirmed that E2F3 combined the promoter of FGF2, and stiffness promoted FGF2 expression. On a stiff matrix, HCC cells cultured with conditioned media (CM) from HSCs increased HCC cells growth and metastasis by binding FGFR1 to activate PI3K/AKT and MEK/ERK signaling pathways. Moreover, conditional E2F3 knockout mice were subjected to CCl4 treatment to assess the role of E2F3 in HSC activation. Additionally, the DEN-induced HCC model was also used to evaluate the role of E2F3 in liver fibrosis and HCC growth. In conclusion, we demonstrated that stiffness-induced HSC activation by E2F3 dependent. Stiffness activated CD36-AKT-E2F3 signaling and targeted FGF2 transcription, subsequently, activated HCC growth and metastasis by FGFR1-mediated PI3K/AKT and MEK/ERK signaling.

## Introduction

Hepatocellular carcinoma (HCC) is the third most common cause of cancer-related death worldwide [1]. Most clinical data indicated that the majority (~80%) of HCC patients have liver cirrhosis backgrounds [2]. Recently, the stiffness measurement involving ultrasound elastography (FibroScan) has been identified as a non-invasive predictor in fibrosis diagnosis and assessment of HCC development [3]. Although the great correlation between matrix stiffness and HCC has been focused in past decades, however, the detailed mechanism by which substrate stiffness promoted HCC initiation and progression still remains largely unknown.

Matrix stiffness is a critical physical attribute of a solid extracellular matrix (ECM). Hepatic stellate cells (HSCs) contribute to the desmoplastic of the liver by differentiating into myofibroblasts (MFs) that deposit ECM [4]. On the contrary, ECM-mediated stiffness promotes malignancies by modulating cell growth, motility, proliferation, metabolism, and metastasis, such as clustering of integrin-based adhesion proteins into focal adhesions, Rho-ROCK activation, and nuclear localization of transcriptional regulators YAP and SNAIL1 [5]. Inhibiting matrix stiffness reduces tumor incidence and improves treatment. Activated HSCs in the liver microenvironment regulate HCC growth by paracrine mechanisms, including secreting growth factors, ECM, cytokines [6, 7]. In response to mechanical stimulation such as stiffness of ECM can induce HSCs to differentiate into cancer-associated fibroblasts (CAF) [8]. CAFs exhibit migratory and contractile properties of MFs and release collagen and chemokines into tumor stroma. Mechanistically, HSCs/MFs transdifferentiation, which means HSCs activation, demands cell surface receptor activation, signal transduction, and gene transcription [9]. Despite the important contributor of activated HSCs to desmoplasia of HCC, it’s still unclear that stiffness leads to mechanosignal transduction in HSCs, which promotes HCC growth in part through secretion of the signaling protein. Therefore, in the present study, we focused on how stiffness-regulated HSCs activation, triggering enhanced proliferation and survival of HCC cells.

The E2F family of transcription factors is essential for cell proliferation, cycle, differentiation, and apoptosis [10]. Accumulating evidence suggests that E2Fs are capable of regulating a large number of genes. E2F3, as a member of the E2F family, was originally identified as an E2F1 paralog through low-stringency hybridization experiments [11]. E2F3 plays important role in animal development. E2F3 acts as a transcriptional activator and increases cellular proliferation through the G1/S transition. Recent studies indicate that E2F3 modulates the expression of several genes, including CDK1 and Survivin. However, the specific target genes of E2F3 in HSC have not been investigated in detail. Recently, E2F3 complexes were involved in TGF-β-induced effects in human myeloid leukemia cells. TGF-β/SMAD-dependent signaling was a classical pathway implicated in liver fibrogenesis. Since the role of E2F3 in HSCs remains unknown, we explored a novel E2F3-dependent mechanism for stiffness-mediated HSC activation.

In the present research, we developed a modular polyacrylamide substrate gel to culture HSCs. We demonstrated that substrate stiffness-induced MF activation of HSCs by activating a CD36-AKT-E2F3 mechanotransduction pathway. This signaling induced E2F3 nuclear targeting, leading to transcription of FGF2, which is critical for HCC growth. In addition, CM of HSCs on a stiff substrate promoted HCC growth in vitro and in vivo compared to that of a soft substrate. Cre-loxP-mediated E2F3 gene deletion in activated-HSC-MFs inhibited HCC growth in mice and CCl4-induced HSC activation and liver fibrosis. Therefore, we explored the substrate stiffness effect on HSCs activation by mechanotransduction properties and to promote HCC growth, which facilitates further studies of targeting matrix stiffness toward the clinical intervention of HCC.

## Materials and methods

### Cell culture

Human primary HSCs were purchased from ScienCell (#5300, San Diego, CA, USA) and LX2 cells (Shanghai Institute of Biochemistry and Cell Biology, Shanghai, China). Hep3B cells were obtained from the American Type Culture Collection (Manassas, VA, USA). All cells were maintained in DMEM (Invitrogen, Carlsbad, USA) containing 10% FBS (Gibco, GrandIsland, USA) at 37 °C with 5% CO2.

### Polyacrylamide hydrogels with incremental stiffness

Polyacrylamide hydrogels with stiffness were prepared as reported previously [12, 13].

### Coinjection of Hep3B and HSC conditioned medium (CM) into mice

Hep3B cells serum-starved for 24 h were collected, each group contain 5 × 10^6^ cells. After centrifugation, each cell pellet was resuspended in 1 ml each of the following CMs: CM of HSCs on 1 kPa, CM of HSC-NT shRNA on 32 kPa, and CM of HSC-E2F3 shRNA on 32 kPa. Hep3B cells were then incubated with a CM at 37 °C for 2 h, Hep3B resuspended with the CM were co-injected into SCID mice subcutaneously (each injection contained 200 µl mixture, 1 × 10^6^ Hep3B cells). Mice were killed at day 10 and tumor volume was calculated by the equation: volume = (length × width2)/2.

### Western blot analysis

We separated proteins by SDS–PAGE and transferred proteins to PVDF membranes. A detailed experiment was performed similarly to previously reported [14, 15].

### Immunofluorescence (IF) and confocal microscopy

Cells on hydrogels were fixed with 4% paraformaldehyde and permeabilized with 0.2% Triton X-100/PBS for 3 min before IF. IF performed with cryosections (7 µm) was done as we have previously described [16]. Confocal microscopy was performed using a confocal laser scanning microscope (LSM 510, Carl Zeiss, Germany) and signals were quantified using the ImageJ software.

### Enzyme-linked immunosorbent assay (ELISA)

Conditioned media (CMs) of HSCs on different substrates were collected and stored at 4 °C. FGF2 concentration of CMs was quantitated using a Human FGF2 Quantikine ELISA Kit (R&D Systems, Inc.) according to the manufacturer’s instructions.

### Cell proliferation assay

Cell Counting Kit-8 (CCK8) reagents (Dojindo, Kumamoto, Japan) were carried as described previously [15].

### Patients’ tissues and cell culture

Patients’ tissues and non-tumor tissues were obtained from our hospital after informed consent was obtained from all patients. All patients didn’t receive any therapy including radiotherapy, chemotherapy, or radiofrequency ablation before surgery.

### CCl4-induced fibrosis mouse model

CCl4 treatment in mice was approved by the Animal protocols and was approved by the Institutional Animal Care and Use Committee of Xi’an Jiaotong University. CCl4 (0.5 mg/kg) diluted in olive oil was administered to age-matched male E2F3 + / + cre and E2F3 F/F cre mice by intraperitoneal injection twice a week for 6 weeks. E2F3 F/F mouse line and collagen1A1-cre transgenic line mice were purchased from Cyagen (Guangzhou, China). The livers of mice were then isolated for H&E, Sirius Red staining, WB, and cryosectioning for IF. The areas stained by picrosirius red were quantitated using the ImageJ software in ten randomly chosen microscopic fields per slide.

### Statistical analysis

Results are managed as the mean ± SD and analyzed by SPSS software, 16.0 (SPSS, Chicago, USA). The statistical approaches mainly included a two-tailed Student’s *t*-test or ANOVA. The difference with *P* < 0.05 was regarded to be significant. Graphs were mainly made by GraphPad Prism 6 (GraphPad, San Diego, USA).

## Results

### Increasing matrix stiffness-induced MF activation of HSCs and promoted the expression of E2F3 in human HSCs and LX2 cells

**Fig. 1:**
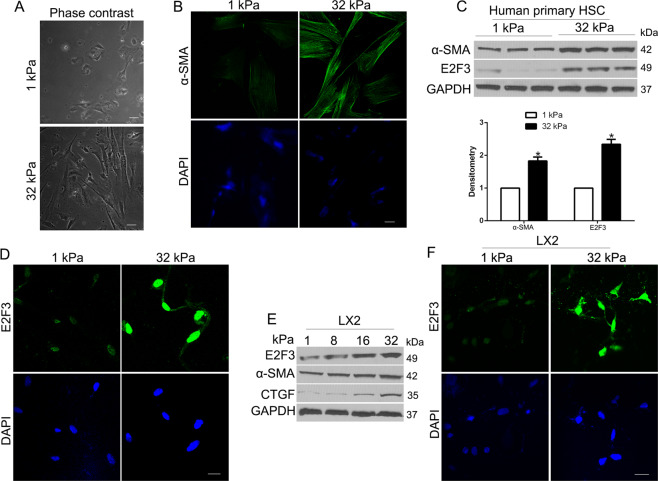
Substrate stiffness promotes E2F3 expression and MF activation of HSCs. **A** HSCs were plated on a 1 or 32 kPa hydrogel and subjected to phase-contrast microscopy. **B** IF for α-SMA detected HSC activation-induced by increasing stiffness. **C** HSCs were plated on a 1 or 32 kPa hydrogel and subjected to WB with quantitative data shown. 32 kPa stiffness promoted HSC activation and E2F3 protein level. *P < 0.05 by *t*-test, *n* = 3 repeats. **D** IF assay was used to analyze stiffness-induced E2F3 nuclear expression. Cell nuclei were counterstained with DAPI. **E** LX2 cells plated on polyacrylamide hydrogels were collected for WB. **F** IF revealed nuclear expression of E2F3 in LX2 cells on 32 kPa. Cell nuclei were counterstained with DAPI.

Pathological fibrosis is driven by a feedback loop in which the fibrotic extracellular matrix is both cause and consequence of fibroblast activation [17]. We used different stiffness polyacrylamide hydrogels to culture HSCs to confirm whether matrix stiffness-regulated HSC activation. We demonstrated that the cell morphology of HSCs seeded on hard 32 kPa hydrogel showed an elongation, protrusive cell morphology, character of activated HSCs, while cells on 1 kPa soft gel showed a round and circularity morphology (Fig. 1A), which was consistent with the phenomenon in rat HSCs [18]. Moreover, cells on 32 kPa presented a strong α-SMA, a marker of activated-HSCs compared to 1 kPa (Fig. 1B). Since stiffness activated fibroblasts independent of TGF-β signaling, we explored a novel pathway for stiffness-mediated HSC activation. We confirmed that E2F3, a transcription factor of cells, was enhanced by 32 kPa stiffness compared to 1 kPa by WB and IF (*P* < 0.05 by *t*-test, Fig. 1C, D), which identified E2F3 as a mechanosensitive mediator in mechanical stiffness-induced HSCs activation. To confirm this, we facilitated immortalized HSCs LX2 cells on incremental stiffness gels. We found that the E2F3 expression level increased with the stiffness, as well as the expression of α-SMA and CTGF, markers of HSC activation (Fig. 1E). IF also confirmed that stiffness gel increased E2F3 expression in LX2 cells (Fig. 1F). Taken together, these results confirm that E2F3 is a mechanotransducer of mechanical stimulation-induced HSCs activation.

### E2F3 knockdown abolished the matrix stiffness-induced HSCs activation

**Fig. 2:**
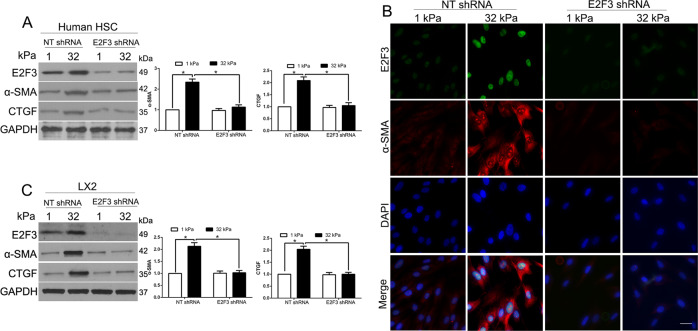
Stiffness induces HSC activation by an E2F3-dependent mechanism. **A** Primary human HSCs on 1 or 32 kPa were transduced with lentiviruses encoding NT shRNA or E2F3 shRNA. Stiffness-mediated HSC activation was abrogated by E2F3 knockdown. *P < 0.05 by ANOVA, *n* = 3. **B** Primary HSCs seeded on a 1 or 32 kPa gel were transduced with NT shRNA or E2F3 shRNA for IF. Stiffness-mediated upregulation of α-SMA and E2F3 was abrogated by E2F3 gene deletion. Cell nuclei were counterstained by DAPI. **C** WB revealed that stiffness-mediated HSC activation was abrogated by E2F3 gene deletion. *P < 0.05 by ANOVA, *n* = 3 repeats.

We first used lentiviruses encoding non-targeting shRNA (NT shRNA) or E2F3 shRNA to knock down E2F3 in HSCs on different gels. WB showed that E2F3 knockdown suppressed the stiffness-induced increase of α-SMA and CTGF in human HSCs (*P* < 0.05 by ANOVA, Fig. 2A). As revealed by α-SMA/E2F3 double IF (Fig. 2B), 32 kPa stiffness-induced E2F3 and α-SMA-positive overexpression, and these effects were abolished in E2F3 knockdown cells. Moreover, WB confirmed that stiffness-mediated HSC activation was inhibited in E2F3 knockdown LX2 cells (*P* < 0.05 by ANOVA Fig. 2C). Therefore, E2F3 was required and necessary for stiffness-induced HSCs activation.

### Stiffness promoted E2F3 expression is mediated by CD36

**Fig. 3:**
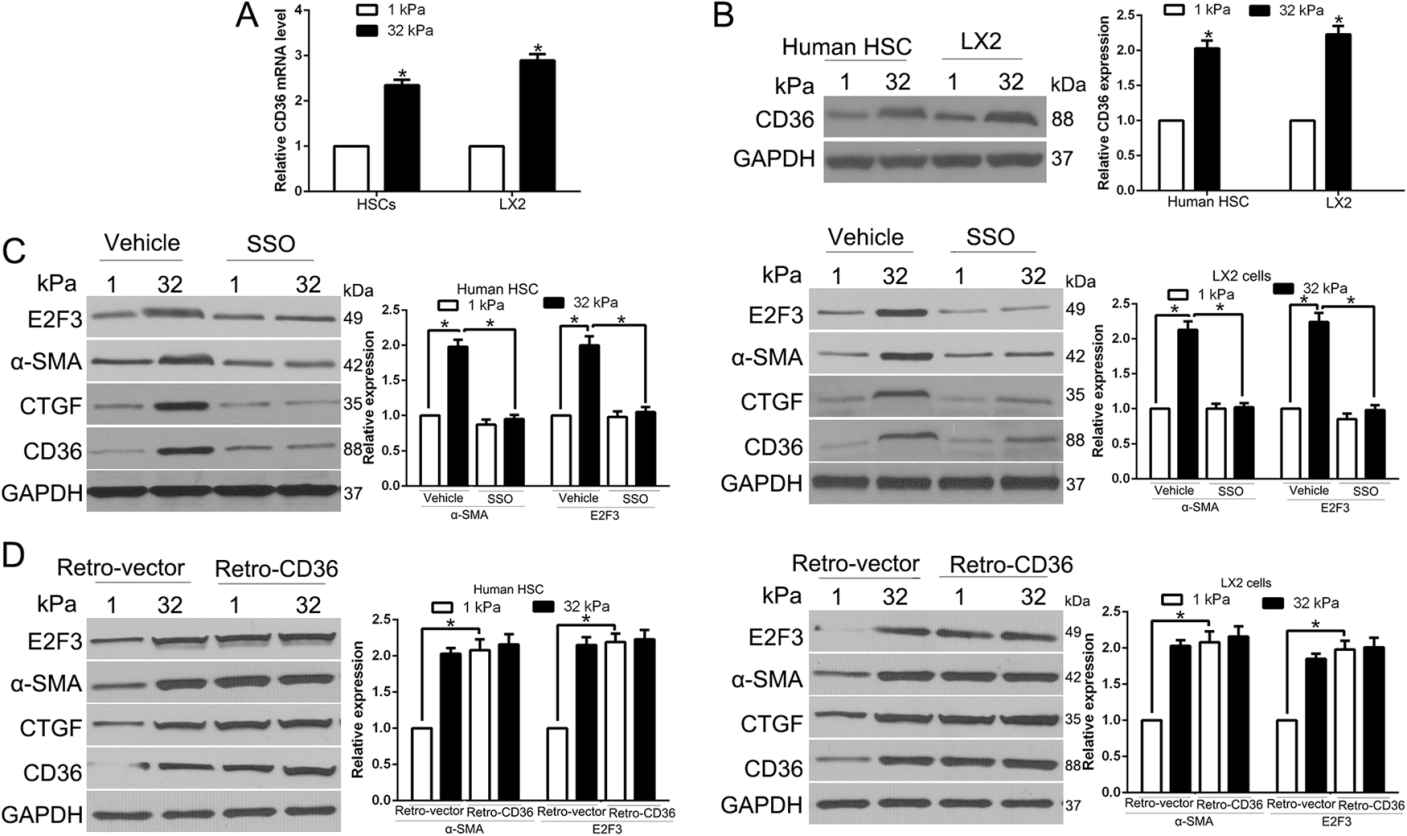
Stiffness promoted E2F3 expression is mediated by CD36. **A** Quantitative real-time PCR after reverse transcription revealed that stiffness significantly increased CD36 mRNA level (*P* > 0.05 by *t*-test, *n* = 6). **B** Primary human HSCs and LX2 cells on hydrogels were collected for WB. CD36 protein was increased by stiffness. *P < 0.05 by ANOVA, *n* = 3 for WB. **C** Primary human HSCs or LX2 cells on 1 or 32 kPa were treated with vehicle or CD36 inhibitor SSO. Stiffness-mediated E2F3 upregulation and HSC activation was abrogated by SSO. *P < 0.05 by ANOVA, *n* = 3 for WB. **D** Primary human HSCs or LX2 transduced with retroviruses encoding LacZ (Retro-vector) or CD36 (Retro-CD36) were seeded on 1 or 32 kPa for WB. CD36 led to HSC activation, E2F3 upregulation in HSCs on 1 kPa. *P < 0.05 by ANOVA, *n* = 3 repeats.

Mechanistically, HSCs activation requires cell surface receptor activation, and intracellular signal transduction, which combined ultimately modulate distinct gene expression profiles to maintain a new phenotype. We performed qRT-PCR and WB and found that CD36 mRNA and protein level all increased in primary human HSCs and LX2 cells (*P* < 0.05 by *t*-test, Fig. 3A, B). Moreover, CD36 expression in HSCs was confirmed by immunofluorescence (Fig. S1). To confirm that the role of CD36 in stiffness-induced E2F3 overexpression, we first used sulfo-*n*-succinimidyl oleate (SSO, 200 μM), an irreversible inhibitor of CD36, to inhibit CD36 activity. Intriguingly, stiffness-induced overexpression of α-SMA and E2F3 protein levels was markedly inhibited by SSO in both human HSCs and LX2 cells (*P* < 0.05 by ANOVA, Fig. 3C). In addition, we transfected cells by control or CD36 retroviral transduction vectors and cultured cells on different gels. CD36 overexpression in cells on 1 kPa indeed upregulated α-SMA and E2F3 expression, similar to the effects of 32 kPa stiffness (*P* < 0.05 by ANOVA, Fig. 3D). Therefore, CD36 is pivotal for stiffness-mediated E2F3 expression and HSC activation.

CD36 is a transmembrane protein involved in the transport and metabolism of certain lipids, such as long-chain fatty acids and oxidized low-density lipoproteins. For whether fatty acids metabolism was involved in the matrix stiffness-regulated p-AKT and E2F3 expression, we performed WB and showed that the fatty acid synthase (FASN) was significantly increased in 32 kPa stiffness compared in 1 kPa stiffness (*P* < 0.05, Fig. S2A), which suggested that high stiffness accumulated more fatty acids. FASN, the key enzyme of fatty acids synthesis, catalyzes the synthesis of fatty acids from acetyl CoA and malonyl-CoA. Thus, we knock down the expression of FASN in HSCs by siRNA (Fig. S2B). FASN knockdown reduced and production of fatty acids in HSCs, and then decrease the lipid metabolites downstream of fatty acids. In addition, stiffness-induced overexpression of p-AKT and E2F3 protein levels was markedly inhibited by FASN siRNA in HSCs (Fig. S2B).

### Stiffness promoted E2F3 expression by activating a CD36-AKT pathway

**Fig. 4:**
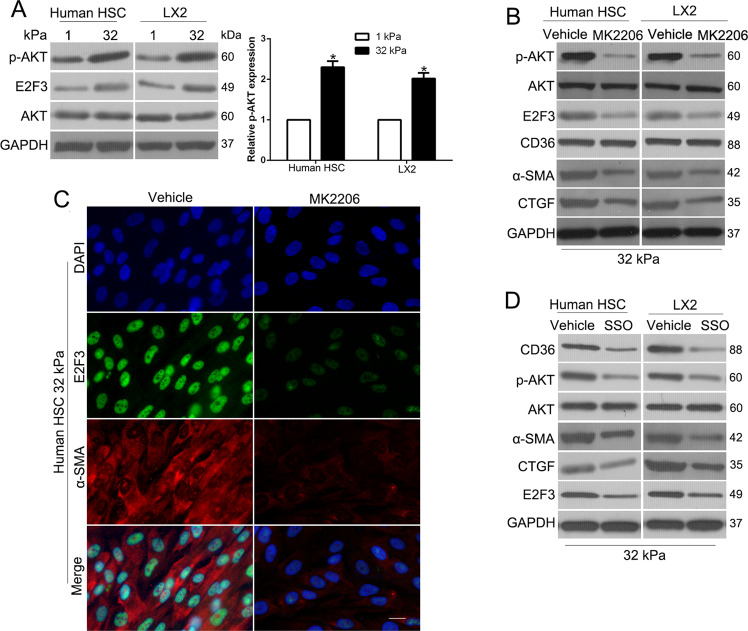
Stiffness promoted E2F3 expression by activating a CD36-AKT pathway. **A** WB revealed that stiffness-induced AKT phosphorylation at S473. **B** AKT inhibitor MK2206 reduced p-AKT(S473), total E2F3, and HSC activation markers, α-SMA, CTGF level of HSCs. Data represent multiple repeats with similar results. **C** IF showed that MK2206 suppressed stiffness-mediated E2F3 overexpression and activation of HSCs. **D** CD36 inhibitor SSO reduced p-AKT, and E2F3 levels of HSCs.

To conjunct the cell surface receptor CD36 to signal transduction to E2F3 expression, we performed WB to confirm that AKT phosphorylation was intermediate according to previous studies (*P* < 0.05 by *t*-test, Fig. 4A) [19]. To facilitate if stiffness-induced E2F3 expression was mediated by AKT, we treated cells with AKT phosphorylation inhibitor MK2206 and found that MK2206 reversed stiffness-induced E2F3 expression and HSC activation (Fig. 4B, C). Moreover, we treated cells with SSO and results showed that SSO reduced stiffness-induced AKT phosphorylation and E2F3 expression (Fig. 4D), which revealed that CD36 is required for stiffness-induced AKT phosphorylation and E2F3 upregulation. Taken together, stiffness promoted E2F3 overexpression and HSC activation by activating a CD36-AKT-E2F3 mechanosensitive signaling pathway of HSCs.

### Stiffness-E2F3 axis promotes transcription of tumor-promoting factors of HSCs

**Fig. 5:**
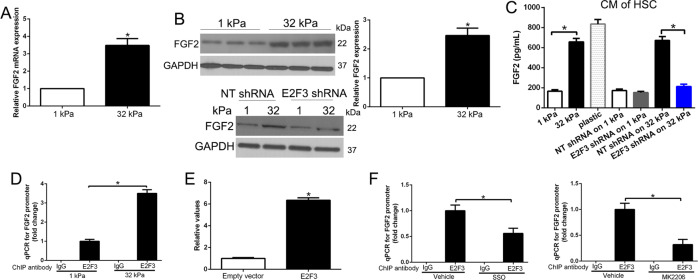
Stiffness-E2F3 axis promotes transcription of tumor-promoting factors FGF2 of HSCs. **A** Stiffness increased FGF2 mRNA as revealed by qPCR after reverse transcription. *P < 0.05 by *t*-test, *n* = 6. **B** Stiffness increased FGF2 protein through HSC E2F3. *P < 0.05 by *t*-test, *n* = 3. **C** Conditioned medium (CM) of HSCs on different substrates were collected for ELISA to quantitate HSC-released FGF2. Stiffness promoted HSCs to release FGF2 through HSC E2F3. *P < 0.05 by ANOVA, *n* = 6. **D** E2F3/DNA complexes were pulled down by control IgG or anti-E2F3 for ChIP-qPCR and qPCR was performed with primers for FGF2 promoter. The stiffness increased E2F3 on the FGF2 promoter. *P < 0.05 by ANOVA, *n* = 3. **E** Luciferase promoter assays in HEK-293A cells of the FGF2 promoter reveal that the addition of an activating E2F3 induces an obvious increase of FGF2 promoter activity. **F** SSO or MK2206 reduced E2F3 on FGF2 promoter of HSCs. *P < 0.05 by ANOVA, *n* = 3.

Our studies had performed RNA sequence to explore that stiffness-E2F3 regulated transcription of HSCs. Moreover, we identified different pathways involved in stiffness-induced HSC activation. Stiffness promoted transcription of more than 20 tumor-promoting factors, including FGF, CTGF, and IL6 [12]. Since FGF2 regulates cancer growth and metastasis, including HCC, lung cancer, breast cancer [20–22], we used FGF2 as a molecular to confirm the hypothesis. Intriguingly, qRT-PCR confirmed that FGF2 mRNA level was increased by stiffness and WB showed that stiffness upregulated FGF2 protein level depending on HSC E2F3 expression (*P* < 0.05 by *t*-test, Fig. 5A, B). Moreover, ELISA detected that FGF2 concentration in the culture medium was increased by stiffness-E2F3 axis (Fig. 5C). Therefore, stiffness-E2F3 promoted HSCs to generate and release tumor-promoting factors, FGF2. To confirm that E2F3 regulated FGF2 expression by directly combined FGF2 gene promoter, we used CHIP-qPCR to analyze stiffness increased E2F3 on FGF2 promoter (*P* < 0.05 by ANOVA, Fig. 5D). To further determine the FGF2 promoter is E2F3 responsive, the FGF2 reporter construct was cotransfected with expression vectors for E2F3 in HEK-293A cells, E2F3 induced a sixfold activation, suggesting that E2F3 was an activator of FGF2 transcription (Fig. 5E). Furthermore, SSO or MK2206 reduced FGF2 promoter E2F3 expression (*P* < 0.05 by ANOVA, Fig. 5F). In conclusion, the stiffness-E2F3 axis promoted FGF2 gene transcription of HSCs.

### Stiffness-E2F3 pathway promotes paracrine tumor-promoting effects of HSCs in vitro and in vivo

**Fig. 6:**
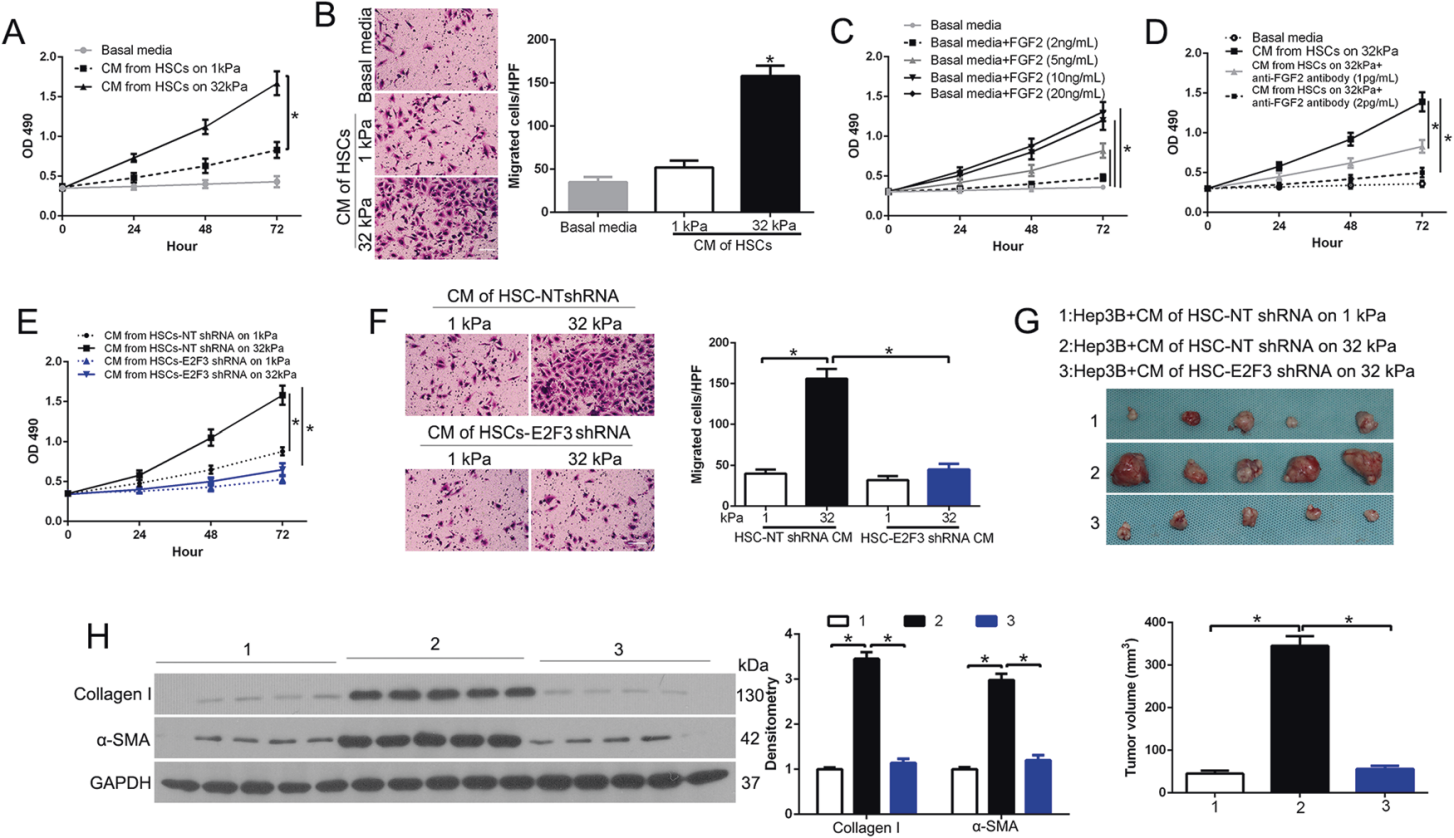
Stiffness potentiates tumor-promoting effects of HSCs through HSC E2F3. **A** HSC conditioned media (CMs) were used as stimulants for Hep3B proliferation assays. HSC 32 kPa CM promoted Hep3B proliferation as compared to 1 kPa CM. *p < 0.05 by ANOVA, *n* = 6. **B** HSC CMs were used as attractants for Hep3B cells in the Transwell assay. CM of HSCs on 32 kPa promoted Hep3B migration as compared with. that of HSCs on 1 kPa. *p < 0.05 by ANOVA, *n* = 3. **C** Recombinant FGF2 dose-dependently promoted Hep3B proliferation. *p < 0.05 by ANOVA, *n* = 4. **D** CM of HSCs on 32 kPa promoted Hep3B proliferation and this effect of 32 kPa CM was dose-dependently reduced by a neutralizing anti-FGF2 antibody. *p < 0.05 by ANOVA, *n* = 3. **E** The effects of HSC CM on Hep3B proliferation were abrogated by E2F3 knockdown in HSCs. *p < 0.05 by ANOVA, *n* = 5. **F** CMs were used as attractants in the Transwell assay. CM of control HSCs on 32 kPa promoted Hep3B cell migration as compared to that of control HSCs on 1 kPa, and this effect of 32 kPa CM was abrogated by E2F3 knockdown in HSCs. *p < 0.05 by ANOVA, *n* = 6. **G** Serum-starved Hep3B pretreated with a CM at 37 °C for 2 h were resuspended in the CM and Hep3B/CM were then co-injected into SCID mice subcutaneously. Tumor nodules were isolated and quantitated on day 10. *P < 0.05 by ANOVA, *n* = 5 per group. **H** Tumor lysates were subjected to WB for α-SMA and Collagen I. *P < 0.05 by ANOVA, *n* = 3.

To confirm that conditioned medium (CM) of HSCs promoted HCC growth, we collected CM from HSCs on gels for CCK8 and Transwell assay to investigate the role of Hep3B cell proliferation and migration. Amazingly, CM of HSCs on 32 kPa promoted Hep3B proliferation and migration as compared to 1 kPa (*P* < 0.05 by ANOVA, Fig. 6A, B). Next, we found that recombinant FGF2 dose-dependently promoted Hep3B proliferation (*P* < 0.05 by ANOVA, Fig. 6C), and a neutralizing FGF2 antibody dose-dependently inhibited the effect of 32 kPa CM on Hep3B proliferation (*P* < 0.05 by ANOVA, Fig. 6D). In addition, the effects of 32 kPa CM on Hep3B proliferation and migration were suppressed by E2F3 knockdown in HSCs (*P* < 0.05 by ANOVA, Fig. 6E, F). These data suggest that stiffness-E2F3 increased tumor-promoting effects of HSCs via producing paracrine factors FGF2. To validate stiffness-E2F3 effects in vivo tumor growth, we used a tumor cell/ CM co-implantation mouse model. After Hep3B serum-starved for 2 h, we remixed Hep3B and CM to co-inject mice subcutaneously. Ten days later, we found 32 kPa CM promoted Hep3B growth compared to 1 kPa CM (*P* < 0.05 by ANOVA, Fig. 6G), and this effect was abolished by E2F3 knockdown in HSCs. Furthermore, tumors from 32 kPa CM/Hep3B showed higher α-SMA and Collagen I compared to other groups, which revealed that stiffness facilitated the effects of HSCs on tumor growth-dependent E2F3 of HSCs (*P* < 0.05 by ANOVA, Fig. 6H).

Previous studies confirmed that FGF2 exerts its biological function via combining FGF receptor 1(FGFR1) in lung cancer cells [21]. To determine whether FGF2 could interact with FGFR1, co-IP assays were performed. Anti-FGF2 antibodies, but not control IgG, immunoprecipitated FGFR1 from cell lysates treated with FGF2 (Fig. S3A, left panel). Reciprocally, anti-FGFR1 antibodies could also immunoprecipitated FGF2 from cell lysates (Fig. S3A, right panel). To further evaluate the stiffness-induced FGF2 release, and interact with FGFR1, we performed Co-IP in the cell lysates from cells cultured in CM on gels. Anti-FGF2 antibodies, but not control IgG, could coimmunoprecipitate FGFR1 protein in cell lysates (Fig. S3B). Moreover, we confirmed that FGF2 or CM promoted the activation of PI3K/AKT and MEK1/2/ERK1/2 pathway to potentiate cell growth (Fig. S3C, D). Thus, stiffness-E2F3 axis induced FGF2 release promoted HCC cell growth through FGFR1-mediated PI3K/AKT and MEK1/2/ERK1/2 pathway activation.

### E2F3 is overexpressed in the HSCs/MF murine and the patient’s HCC and CCl4-induced fibrosis was reversed in conditional E2F3 knockout mice

**Fig. 7:**
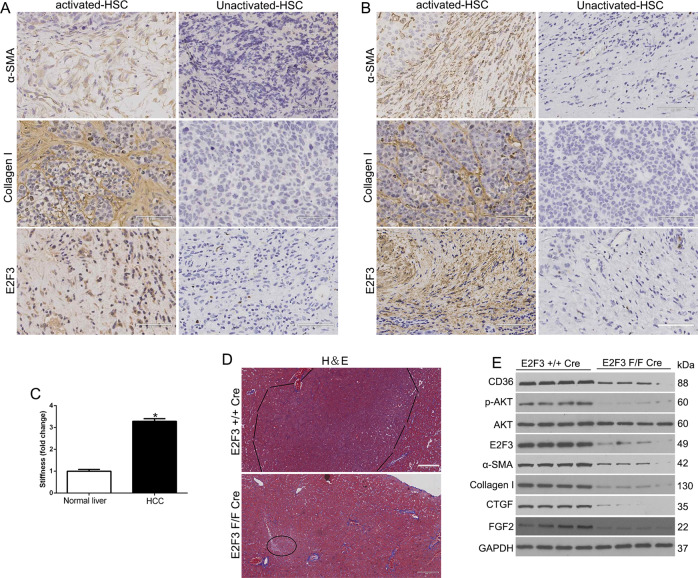
E2F3 is overexpressed in the HSCs/MF murine and patient’s HCC. IHC of α-SMA, collagen, and E2F3 showed strong staining in the activated-HSC/MF surrounding Hep3B/CM (**A**) and patients’ (**B**) tumors. **C** Atomic force microscopy (AFM) was used to measure Young’s modulus of liver cryosections. The stroma of HCC is significantly stiffer than that of normal liver. **D** Representative images of mouse livers from the indicated genotype induced by DEN. **E** WB revealed that CD36 and p-Akt levels of E2F3 F/F Cre HCC were reduced as compared to those of E2F3 + / + Cre HCC.

**Fig. 8:**
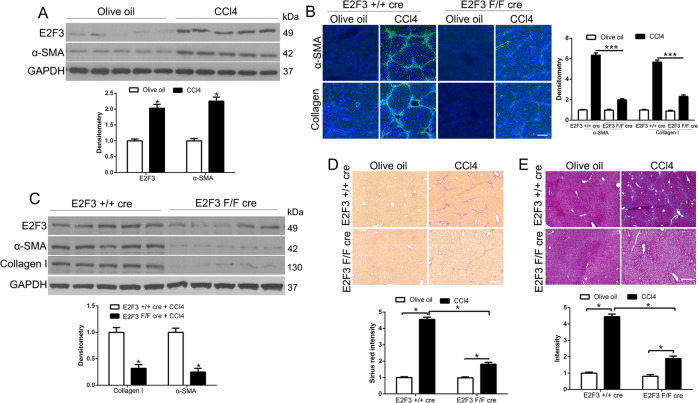
CCl4-induced HSC activation and liver fibrosis are impaired in E2F3 F/F cre mice as compared to E2F3 + / + cre mice. **A** Livers of olive oil-treated mice or CCl4-treated mice were collected for WB. CCl4 treatment upregulated α-SMA and E2F3 in murine liver. Densitometry is shown on the bottom. *p < 0.05 by *t*-test. **B** E2F3 F/F cre mice and E2F3 + / + cre mice were treated with CCl4 for 6 weeks and their livers were. collected for IF for HSC activation markers and collagen I. Quantitative IF data are shown on the right. E2F3 F/F cre livers showed reduced α-SMA and collagen I level, as compared to E2F3 + / + cre livers after CCl4 treatment. *p < 0.05 by *t*-test. **C** CCl4-treated livers were subjected to WB and densitometry data were shown on the bottom. CCl4-treated E2F3 F/F cre livers contained reduced α-SMA and collagen I level as compared to E2F3 + / + cre livers. *p < 0.05 by *t*-test. **D**, **E** Sirius red staining and Trichrome staining of liver sections are shown. Collagen deposition was reduced in CCl4-treated E2F3 F/F cre livers as compared to E2F3 + / + cre livers. *p < 0.05 by *t*-test.

We used the subcutaneous Hep3B/CM tissues and the patient’s HCC tissues to explore E2F3 expression in activated-HSC/MF. We found the α-SMA, Collagen, and E2F3 showed strong staining in the activated-HSC/MF surrounding tumors (Fig. 7A, B). Moreover, we detected stiffness of HCC tissues (Young’s modulus) by atomic force microscopy (AFM) and found that the stroma of HCC was significantly stiffer than that of normal liver (*P* < 0.05 by ANOVA, Fig. 7C). To furthermore investigate the role of E2F3 for HCC growth, we cross E2F3 F/F mice with Collagen1A1-cre transgenic mice to conditionally knockout E2F3 in activated-HSC. A single peritoneal injection of DEN at a dose of 20 μg/kg was performed. Mice were randomly sacrificed at 8, 10, and 12 months. Interestingly, the E2F3 + / + cre mice displayed macroscopic tumors compared to E2F3 F/F cre mice (Fig. 7D and Fig. S4). To investigate that cre expression was cell type-specific and it induced E2F3 deletion in certain cell types in the HSCs. This was confirmed by a confocal fluorescence image containing both cre-GFP-negative and -positive cells (Fig. S5). We demonstrated that E2F3 accumulated in the nucleus and α-SMA-positive stress fibers formed in a control HSC but not in E2F3-null HSCs (cre-GFP positive). WB confirmed that α-SMA, Collagen I, FGF2 were down-regulated in E2F3 F/F cre HCC as compared to E2F3 + / + cre HCC (*P* < 0.05 by *t*-test, Fig. 7E). Since CD36/AKT pathway was activated in MFs, we performed WB to demonstrate that CD36 and p-AKT were reduced in E2F3 F/F cre HCC compared to E2F3 + / + cre HCC (*P* < 0.05 by *t*-test, Fig. 7E). In addition, we used CCl4 to induce liver fibrosis and found that E2F3 was increased in CCl4-induced liver, moreover, CCl4-mediated HSC activation was reduced in E2F3 F/F cre livers (*P* < 0.05 by *t*-test, Fig. 8). Taken together, E2F3 was a critical factor in HSCs activation in HCC growth.

## Discussion

Crosstalk between tumor and stromal cells in the tumor microenvironment alters its properties and facilitates the growth, metastasis behavior of tumor cells [23]. However, it’s unclear that the activated HSC/MFs role in tumor growth. The cancer-associated fibroblasts (CAFs) are responsible for the remodeling of ECM and subsequent growth and dissemination of tumor cells [24]. Liver fibrosis, a critical concern caused by a chronic injury, which provides a permissive environment for HCC development, is derived from activated HSCs. In this study, we confirmed that stiffness-induced HSC activation through E2F3 in reciprocity. Stiffness induces E2F3 nuclear transcription of FGF2, a tumor-promoting factor, to promote HCC growth and metastasis (Supplementary Fig. 1E). On a stiffness environment, cell surface receptor CD36 activation, intracellular AKT/E2F3 transduction, FGF2 transcription, and HSCs expressed increased levels of α-SMA, a marker of HSC activation, these combined ultimately maintain a novel phenotype. Since E2F3 governing HSC activation remains poorly understood, represents critical knowledge in this field. Taken together, we demonstrated for the first time that stiffness-induced HSC activation through E2F3, and this microenvironment promoted tumor growth and metastasis.

E2F3, a potent transcriptional inducer of cell-cycle progression, was dysregulated in many cancers. E2F3 regulated genes transited from G1 to the S phase and DNA replication. E2F3 acts as a transcriptional activator and increases cellular proliferation [25]. In this study, we confirm for the first time that E2F3 modulates the expression of FGF2 by binding its promoter. Moreover, we show a novel role of E2F3 in stiffness-induced HSC activation, which provides a new insight to understand the mechanotransduction and activation of HSCs.

Fibroblast growth factor (FGF) plays an important role in cancer development and progression [26]. FGF2 was involved in cell growth, differentiation, and metastasis of cancers, including HCC [27]. In research, we confirmed that FGF2 was a potent stimulator of cell growth, and has a high affinity for binding to FGFR1. FGFR1, an oncogenic receptor tyrosine kinase, plays a fundamental role in the physiological processes and cancer progression. Here, we demonstrated that FGF2/FGFR1 signaling promotes cell growth and metastasis in the activation of downstream pathway PI3K/AKT and MEK/ERK signaling. We confirmed the origin of FGF2 from activation of HSCs-induced by stiffness.

In China, the clinical data revealed that HCC always combined the background of liver fibrosis caused by chronic hepatitis. The underlying mechanisms by which activated HSCs promote HCC migration and invasion may depend on their ECM remodeling and growth factor release. Previous studies confirmed that the fibrotic liver microenvironment promoted HCC growth and metastasis in the mice model. HGF secreted by activated HSCs promoted the growth and invasiveness of HCC in vitro. Here, we showed that the stiffness microenvironment activates a CD36/AKT/E2F3 mechanosignaling to modulate tumor-promoting factor FGF2 expression. Moreover, conditional knockout E2F3 reduced liver fibrosis and HSCs activation. This provides novel links between the fibrotic microenvironment and HCC. Our data reveal a novel role for E2F3 in HSCs activation and links to HCC growth, which suggests E2F3 as a potential target for HCC treatment.

## Supplementary information

Supplementary legends

Supplementary Figure 1

Supplementary Figure 2

Supplementary Figure 3

Supplementary Figure 4

Supplementary Figure 5

checklist

Corresponding author changes

## Data Availability

All data generated or analyzed during this study are included in this published article.
