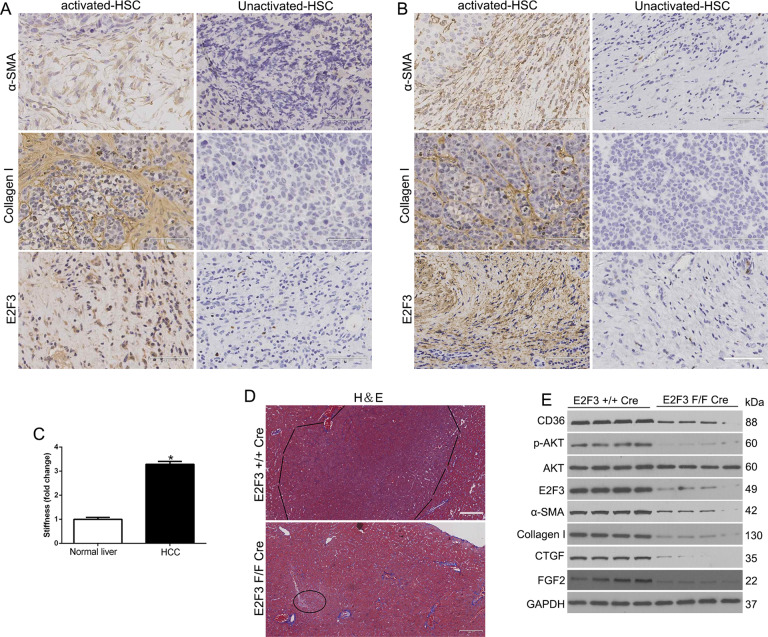# Correction to: Matrix stiffness modulates hepatic stellate cell activation into tumor-promoting myofibroblasts via E2F3-dependent signaling and regulates malignant progression

**DOI:** 10.1038/s41419-022-04528-y

**Published:** 2022-01-28

**Authors:** Zhikui Liu, Huanye Mo, Runkun Liu, Yongshen Niu, Tianxiang Chen, Qiuran Xu, Kangsheng Tu, Nan Yang

**Affiliations:** 1 Department of Hepatobiliary Surgery, the First Affiliated Hospital of Xi’an Jiaotong University, No. 277 Yanta West Road, Xi’an, 710061 China; 2 Key Laboratory of Tumor Molecular Diagnosis and Individualized Medicine of Zhejiang Province, Zhejiang Provincial People’s Hospital (People’s Hospital of Hangzhou Medical College), Hangzhou, Zhejiang 310014 China; 3 Department of Infectious Diseases, the First Affiliated Hospital of Xi’an Jiaotong University, No. 277 Yanta West Road, Xi’an, 710061 China

**Keywords:** Liver cancer, Mechanisms of disease

Correction to: *Cell Death &* *Disease* 10.1038/s41419-021-04418-9, published online 6 December 2021

The original version of this article unfortunately contained a mistake in Fig. 7. The correct figure can be found below. The authors apologize for the error.