# Supplementary material for: Matrix stiffness modulates hepatic stellate cell activation into tumor-promoting myofibroblasts via E2F3-dependent signaling and regulates malignant progression
Source: Cell Death Dis. 2021 Dec 6;12(12):1134. doi: 10.1038/s41419-021-04418-9 (PMC8648844; doi:10.1038/s41419-021-04418-9)
Supplement: Supplementary file 1 — Supplementary legends [file 41419_2021_4418_MOESM1_ESM.docx]

**Figure S1.** The immunofluorescence confirmed the CD36 expression in HSCs.

**Figure S2.** Stiffness promoted FASN expression in HSCs. (A) Primary human HSCs cells on hydrogels were collected for WB. FASN protein was increased by stiffness. (B) Primary human HSCs on 1 or 32 kPa were treated with FASN siRNA. Stiffness-mediated E2F3 and p-AKT upregulation was abrogated by FASN siRNA. * P<0.05.

**Figure S3.** FGF2 combined FGFR1 on HCC cells to activate PI3K/AKT and MEK/ERK signaling. (A) FGF2 treated and (B) HSC conditioned media (CMs) treated HCC cells were performed IP to confirm that FGF2 combine FGFR1. (C) FGF2 treated and (D) HSC conditioned media (CMs) treated HCC cells were performed WB to measure the PI3K/AKT and MEK/ERK signaling. (E) A schematic illustration of stiffness-E2F3 axis induced HCC growth and metastasis.

**Figure S4.** The E2F3 +/+ cre mice displayed macroscopic tumors compared to E2F3 F/F cre mice.

**Figure S5.** Stiffness induces HSC activation by a E2F3-dependent mechanism. (A) Primary murine E2F3 (F/F) HSCs transduced with AdCre-GFP viruses were subjected to IF analysis. A confocal image containing control and E2F3-null cells are shown. Stiffness upregulated E2F3 and α-SMA in a control cell but not in adjacent E2F3-null cells. (B) IF show that Cre-positive HSCs showed no α-SMA staining and the Cre-negative HSCs showed strong α-SMA staining in E2F3 F/F Cre liver tissues.
