# Supplementary figures and images for: Matrix stiffness modulates hepatic stellate cell activation into tumor-promoting myofibroblasts via E2F3-dependent signaling and regulates malignant progression
Source: Cell Death Dis. 2021 Dec 6;12(12):1134. doi: 10.1038/s41419-021-04418-9 (PMC8648844; doi:10.1038/s41419-021-04418-9)

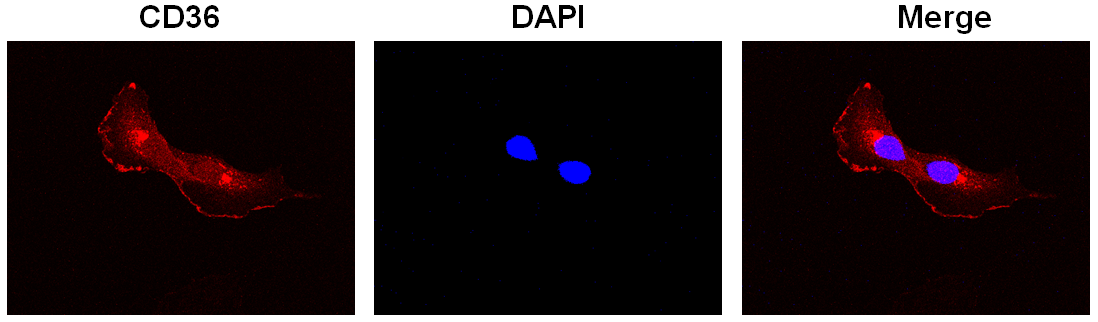

Supplement: Supplementary file 2 — Supplementary Figure 1 [file 41419_2021_4418_MOESM2_ESM.tif]

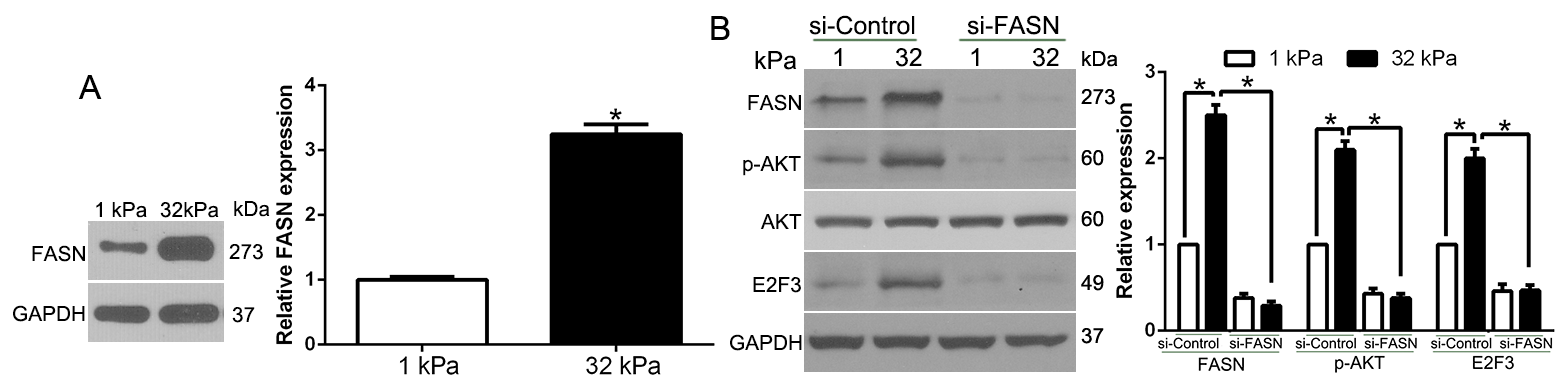

Supplement: Supplementary file 3 — Supplementary Figure 2 [file 41419_2021_4418_MOESM3_ESM.tif]

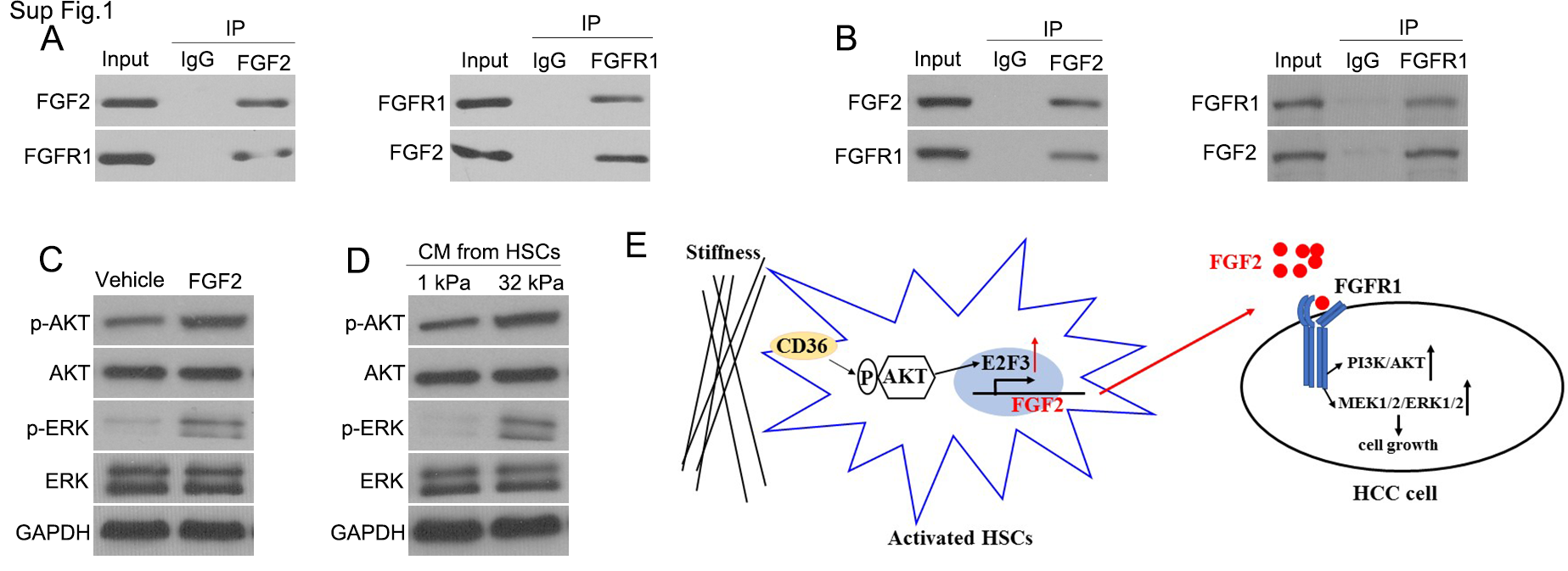

Supplement: Supplementary file 4 — Supplementary Figure 3 [file 41419_2021_4418_MOESM4_ESM.tif]

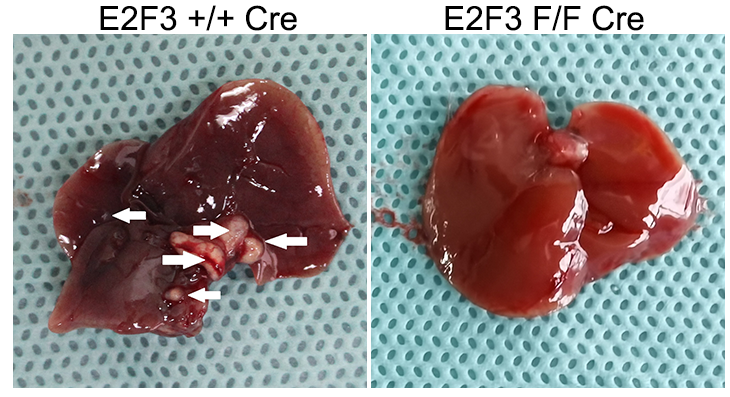

Supplement: Supplementary file 5 — Supplementary Figure 4 [file 41419_2021_4418_MOESM5_ESM.tif]

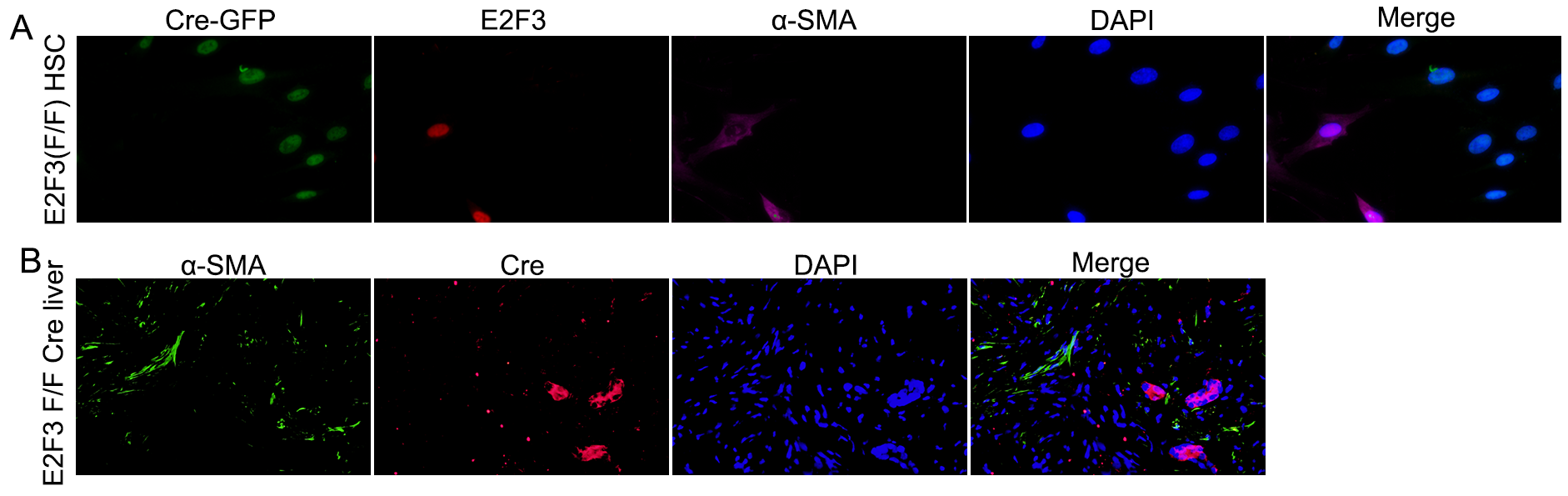

Supplement: Supplementary file 6 — Supplementary Figure 5 [file 41419_2021_4418_MOESM6_ESM.tif]

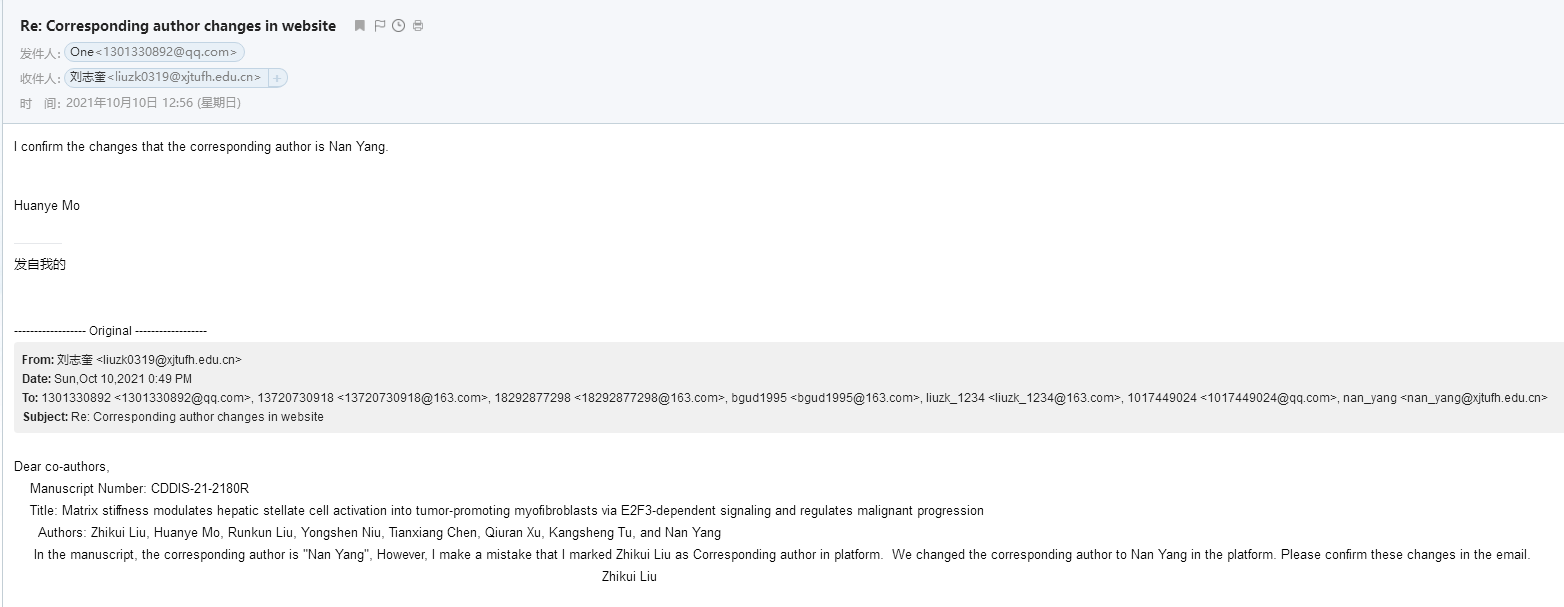


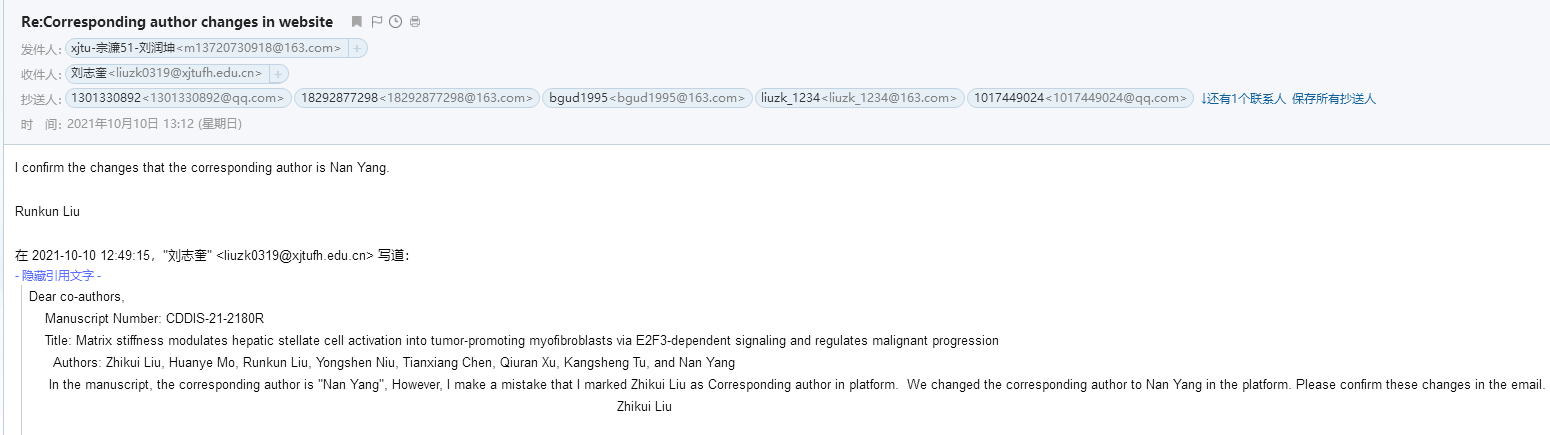


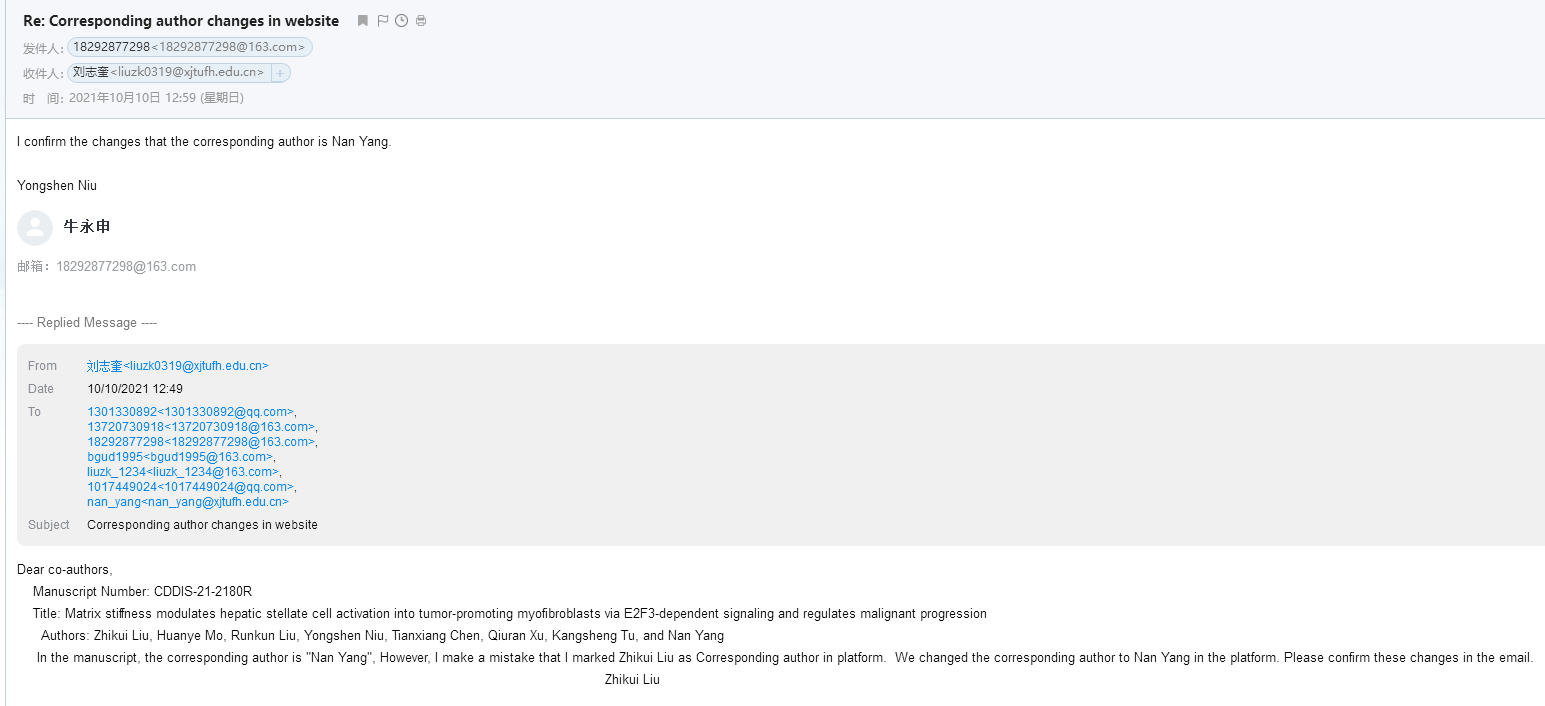


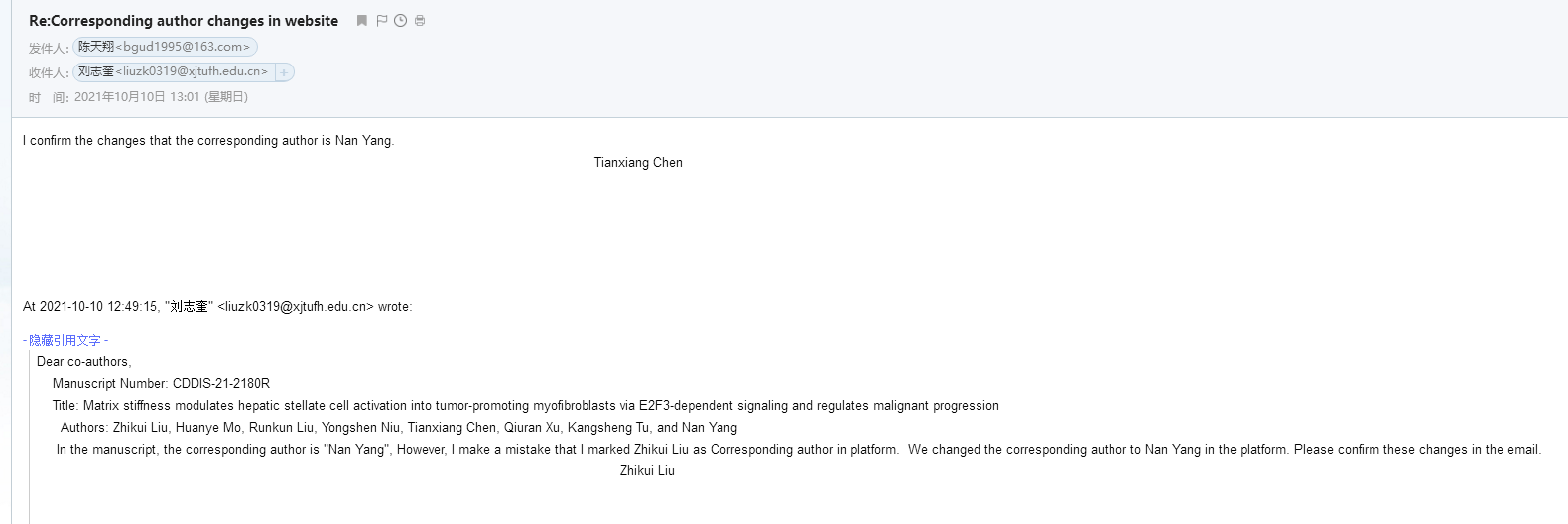


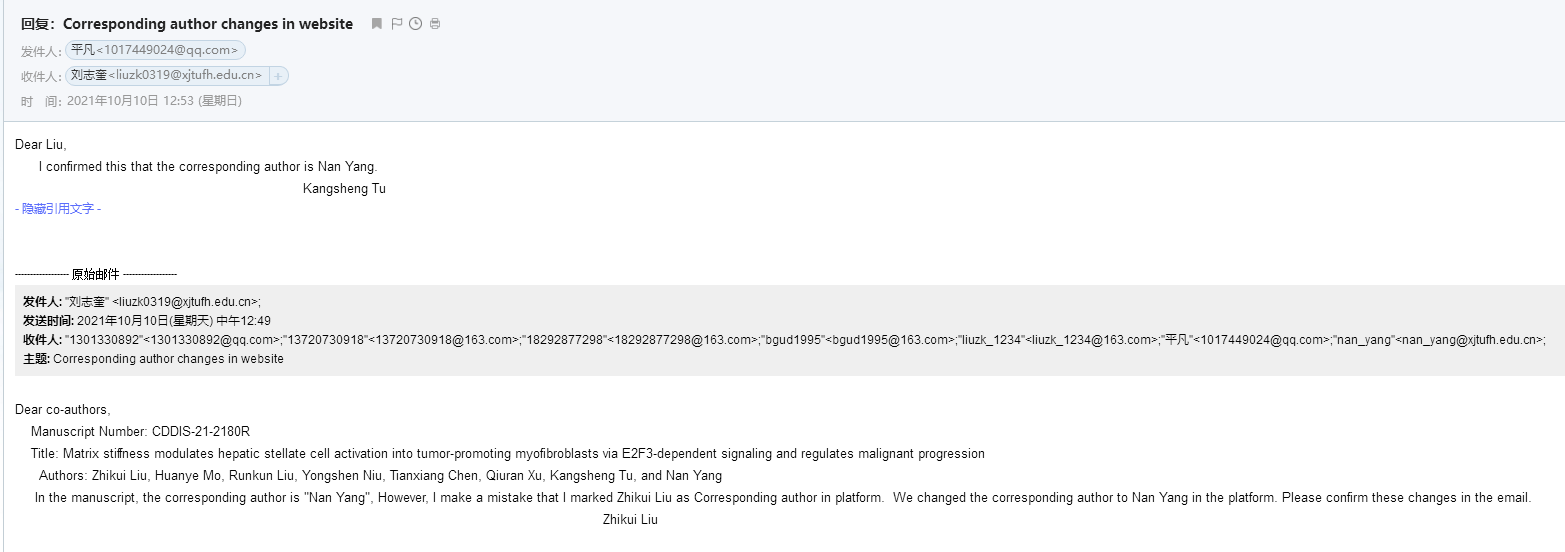


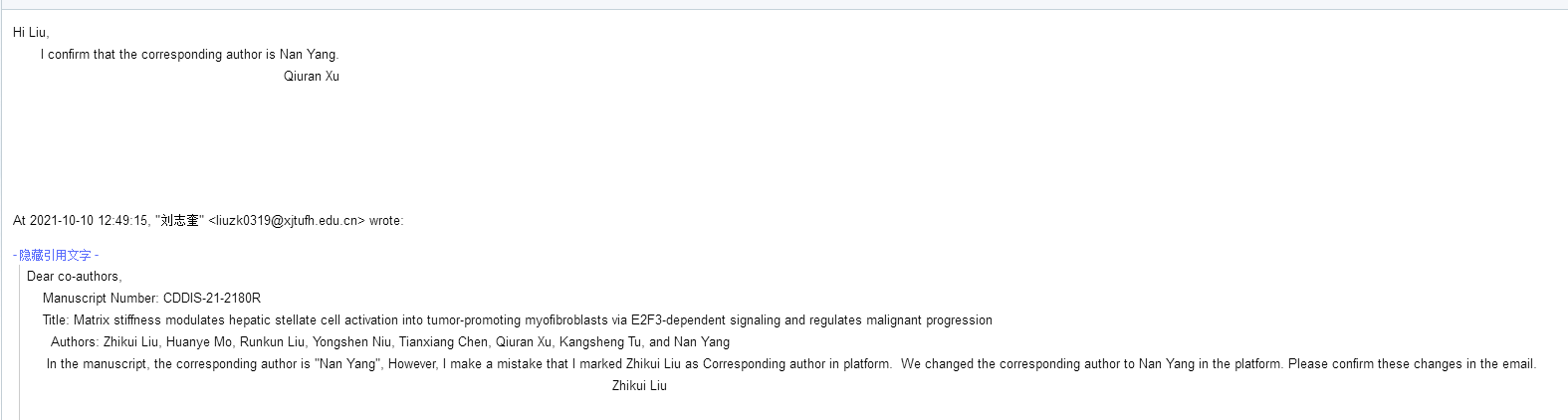


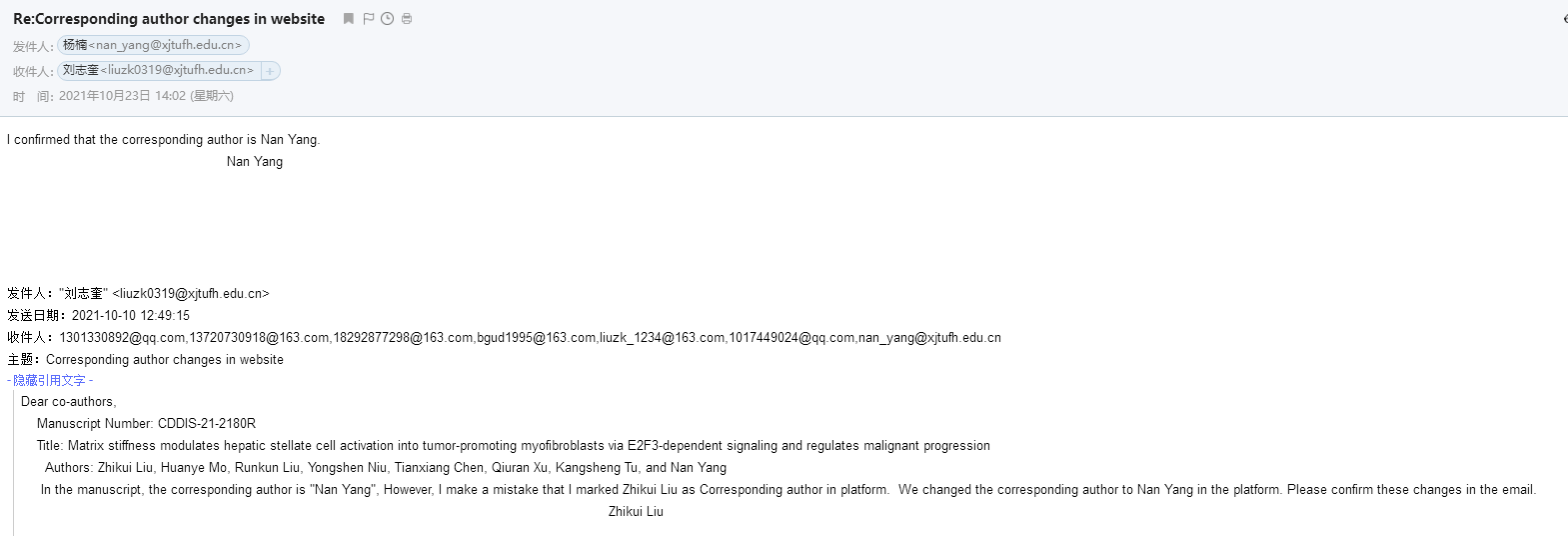

Supplement: Supplementary file 8 — Corresponding author changes [file 41419_2021_4418_MOESM8_ESM.docx]
